# Spatial and multi-level analysis of factors associated with long-acting reversible modern contraceptive use among married women in Ethiopia

**DOI:** 10.1371/journal.pone.0313511

**Published:** 2025-05-28

**Authors:** Bezawit Adane, Bereket Kefale, Elsabeth Addisu, Mastewal Arefaynie, Kefale Mitiku, Yitayish Damtie, Tilahun Degu Tsega, Amare Agmas Andualem, Mahider Awoke Belay, Getachew Tadesse Bedane, Natnael Kebede, Yitbarek Wasihun, Tezera Asfaw, Melaku Yalew

**Affiliations:** 1 Department of Public Health, College of Medicine and Health Sciences, Injibara University, Injibara, Ethiopia; 2 Department of Reproductive and Family Health, School of Public Health, College of Medicine and Health Sciences, Wollo University, Dessie, Ethiopia; 3 Department of Physiology, College of Medicine and Health Sciences, Injibara University, Injibara, Ethiopia; 4 Department of Anesthesia, College of Medicine and Health Sciences, Injibara University, Injibara, Ethiopia; 5 Departments of Statistics, College of Natural Sciences, Wollo University, Dessie, Ethiopia; 6 Department of Health Promotion, School of Public Health, College of Medicine and Health Sciences, Wollo University, Dessie, Ethiopia; 7 Department of Human Anatomy, School of Medicine, College of Medicine and Health Sciences, Wollo University, Dessie, Ethiopia; National Institute of Public Health: Instituto Nacional de Salud Publica, MEXICO

## Abstract

**Background:**

Despite the increasing global use of modern contraceptives, the utilization of Long-Acting Reversible Contraception (LARC) remains low in Sub-Saharan Africa, particularly in Ethiopia, where less than 3% of women use LARC. This low usage is associated with high rates of maternal and under-five morbidity and mortality, contrasting with the global average utilization rate of 15%. Previous studies have found a significant association between marital status and LARC usage, but there is a lack of understanding regarding other individual and community-level factors that may influence this utilization. Additionally, many of the previous studies were either localized or had limited sample sizes, failing to account for spatial variations and clustering effects. In light of these gaps, this study aims to investigate the spatial and multilevel factors from nationally collected data to identify the individual and community-level factors associated with LARC usage among married women in Ethiopia.

**Methods:**

The study employed a cross-sectional design, utilizing secondary analysis of data from the 2019 Ethiopian Mini Demographic and Health Survey (EMDHS). The sample included 5,743 married women; selected using a two-stage stratified sampling technique. Data analysis was conducted with the verification of basic assumptions for multilevel logistic regression. The study utilized spatial and multilevel mixed effect model analysis. To demonstrate the strength and direction of associations, Adjusted Odds Ratios (AOR) with 95% confidence intervals were used.

**Result:**

The study findings revealed significant associations with LARC usage in Ethiopia based on individual-level and community-level variables. Individual-level variables: Older age group was negatively associated with LARC use, (AOR = 0.28, 95%CI = 0.12, 0.64), higher education level was positively associated with LARC use (AOR = 2.99, 95%CI = 1.82, 4.92). Community-level variables: Communities with high female educational status (AOR = 1.75, 95%CI = 1.10, 2.76), communities with higher wealth were also positively associated with LARC use (AOR = 1.92, 95%CI = 1.15, 3.20). Living in Somali region was negatively associated with LARC use, indicating that women residing in this region had a significantly lower likelihood of using LARC (AOR = 0.03, 95%CI = 0.00, 0.33). Overall, these results highlight the influence of both individual-level and community-level factors on LARC utilization among married women in Ethiopia.

**Conclusions:**

Age, educational attainment, community-level female education, community-level wealth, and region were identified as significant predictors of Long-Acting Reversible Contraceptive (LARC) use. Based on these findings, it is recommended that policies be implemented to enhance women’s education across various levels of the population. Special attention should be directed towards the most economically disadvantaged population, and efforts should focus on strengthening the health-seeking behaviors of the people. To improve contraceptive rates, regions with low utilization should be targeted for the expansion of tailored services that align with the lifestyles of their populations.

## Background

Long-acting reversible contraception (LARC) is a highly effective class of contraceptive methods that offer extended birth control without the need for daily maintenance and can be easily reversed upon discontinuation. This category includes intrauterine devices (IUDs) and contraceptive implants [1–3]. In addition to being quite effective, LARC is practical and user-friendly, and it more closely aligns typical use failure rates with perfect use failure rates as compared to shorter-term, user-dependent systems, which raise the likelihood of noncompliance-related method failure [4–6].

Globally, out of 1.9 billion women of reproductive age in 2020, 1.1 billion required family planning, but only 851 million used modern contraceptive methods. The number of women using modern contraceptive family planning increased by 188 million between 2000 and 2020, although significant disparities exist between countries and regions. In sub-Saharan Africa, only 50% of women utilized modern contraception, while 80% of women in industrialized nations used it. This region also faced challenges, with a considerable proportion of teenage girls experiencing unplanned pregnancies, many of whom were married or in a union [7].

Universal access to modern contraception is crucial for all adults and adolescents to prevent unwanted pregnancies and promote satisfying sexual lives while avoiding negative health and social impacts [2]. Family planning services are essential reproductive rights for women and play a vital role in preventing unplanned pregnancies and reducing maternal mortality in low-income countries like Ethiopia [2,3].

Despite the global increase in modern contraceptive utilization, LARC usage remains low in Sub-Saharan Africa, with less than 3% of women using it, leading to higher maternal and under-five morbidity and mortality rates compared to the global average of 15% [8–10]. In Ethiopia, although 41% of married women use modern contraceptives, the utilization of implants and IUDs is only 9% and 2%, respectively, with significant variations among different regions of the country. Unintended pregnancies continue to contribute to maternal and under-five morbidity and mortality, despite various government strategies and emphasis on LARC [3,11,12].

Previous studies in Ethiopia have identified various factors associated with LARC utilization, including age, educational status, family size, religion, place of residence, region, history of abortion, and parity [5,13–18]. However, few studies have explored individual and community-level factors associated with LARC utilization while considering cluster effects from nationally collected data [17,19]. Additionally, many of these studies were local or had small sample sizes, failing to account for spatial variation and clustering effects. Therefore, this study aims to analyze spatial and multilevel factors using nationally collected data, considering both individual and community-level variables associated with LARC use among married women in Ethiopia [18,20].

## Methods

### Study area, period, and design

**Fig 1:**
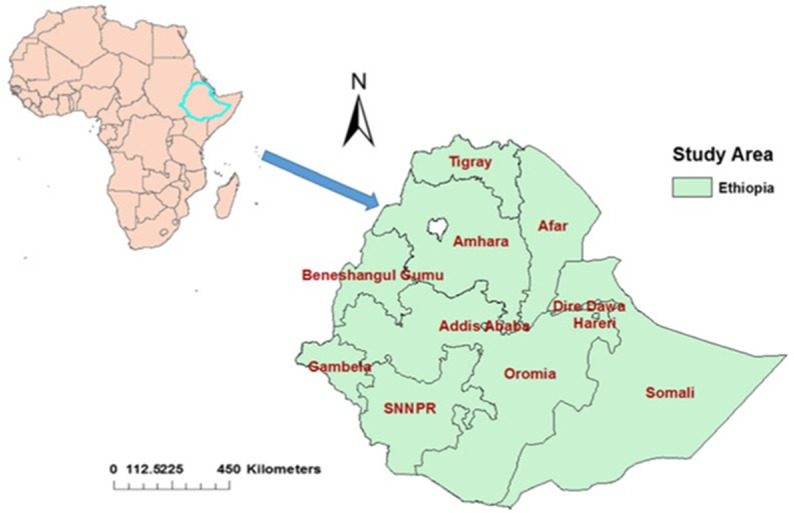
The ArcMap of the study area, Ethiopia in which the study was undertaken (nine geographical regions and two administrative zones).

The study was conducted in Ethiopia, a country situated in Eastern Africa. It spans a total area of 1.1 million square kilometers (Fig 1).

It has a total of 112,100,000 populations, of which 57,120,005 were women [21]. A cross-sectional study design was employed for this research, utilizing secondary analysis of data from the Ethiopian Mini Demographic and Health Survey (EMDHS) 2019 dataset conducted by the Central Statistical Agency (CSA). The survey was carried out over a period of 3 months, from March 2019 to June 2019, and involved a nationally representative sample.

### Sample size determination and sampling procedure/technique

A total of 5,743 married women were included in the study, representing nine geographical regions and two administrative cities in Ethiopia. The sampling frame used for the 2019 EMDHS was derived from the list of all census Enumeration Areas (EAs) created for the 2019 Ethiopia Population and Housing Census (PHC). The census frame consisted of a comprehensive list of 149,093 EAs created for the 2019 PHC. An Enumeration Area typically covered around 131 households on average.

The 2019 EMDHS data has hierarchical type clustering: level 1; married women 15-49 years, level 2; households sampled with in each cluster, level 3;clusters(Primary sampling units typically enumeration areas. The sample was stratified and selected in two stages. Each region was further divided into urban and rural areas, resulting in 21 sampling strata. The dataset also applies stratified two-stage cluster sampling design. It include a cluster ID (v001 or v021), which were used to define clustering during analysis.

**Fig 2:**
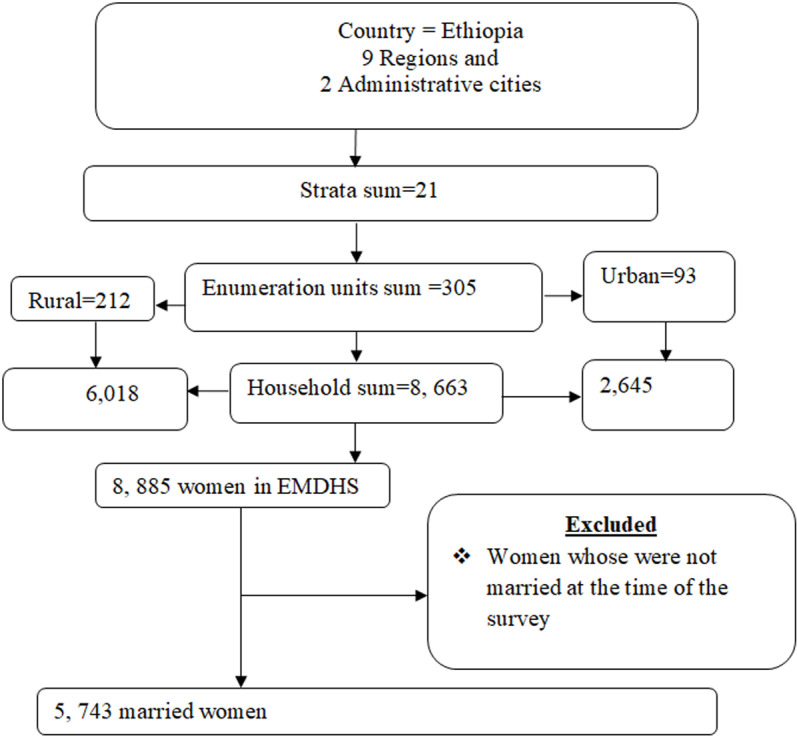
Schematic presentation of sampling procedure spatial and multi-level analysis of factors associated with long-acting reversible modern contraceptive use in Ethiopia.

In the first stage, 305 EAs/clusters (93 urban and 212 rural) were selected using probability proportional to EA size, based on the 2019 PHC frame, with independent selection within each sampling stratum. In the second stage, 30 households per cluster were systematically selected from the updated household listing, ensuring equal selection probability. Overall, the 2019 EDHS sample included 305 clusters, with interviews conducted among 8,885 women aged 15–49 years, of whom 5,743 were married (Fig 2).

### Operational definition

The outcome variable in this study is dichotomized as Long-Acting Reversible Contraceptive (LARC) use, coded as “1” for women who use LARC and “0” for those who do not use it [12]. This variable was generated from a constructed variable in the Ethiopian Demographic and Health Survey (EDHS) data. Women were first asked “Are you or your partner currently doing something or using any method to delay or avoid getting pregnant?” Then, those women who said “yes” were asked again to list the type of method they used and coded based on the type.

### Community-level factors

Community-level variables were created by aggregating individual-level variables. These aggregated variables represent the proportion of a specific variable’s subcategory within a given cluster. To make the aggregate values more manageable, they were grouped based on the national median values. The community-level variables used in the analysis are as follows:

#### Community level of female education.

Aggregated respondent education levels categorized as “Low” (<50%) and “High” (≥50%) education communities [12].

#### Community level of poverty.

Aggregated wealth index categorized as “Low” (<50%) and “High” (≥50%) poverty communities [12].

#### Community media exposure.

Aggregate availability at the cluster level categorized as “Low” (<50%) and “High” (≥50%) communities [12].

### Descriptive and multi-level analysis

The data underwent cleaning to resolve inconsistencies and address missing values. STATA was utilized for recoding, labeling, and exploratory analysis. Descriptive statistics, including frequencies, percentages, tables, graphs, and textual summaries, were used to describe the characteristics of the study participants. Sample weights were applied, and a multilevel analysis was conducted to account for significant intra-cluster correlation (ICC). Given the hierarchical nature of the DHS data, with individuals nested within communities, a two-level logistic regression model was employed to examine the independent effects of the explanatory factors.

Measures of variation, such as ICC, Median Odds Ratio (MOR), and Proportional Change in Variance (PCV), were reported to assess variation between clusters. ICC quantified cluster-level variation, MOR captured unexplained heterogeneity between clusters, and PCV identified the proportion of variance explained by individual- and community-level factors in the multilevel model.

The analysis followed four steps: Model 1 (null model without explanatory variables), Model 2 (adjusted for individual-level factors), Model 3 (adjusted for community-level factors), and Model 4 (adjusted for both individual- and community-level factors). Multi-collinearity among independent variables was assessed using standard errors, with no issues detected. Adjusted Odds Ratios (AOR) with 95% confidence intervals was calculated to determine the strength and direction of associations. The final adjusted model’s adequacy was evaluated through log-likelihood tests comparing it to previous models.

### Spatial analysis

#### Spatial autocorrelation.

To evaluate the degree of clustering of LARC use in the regions/zones, Moran’s spatial autocorrelation method was utilized. The calculation of Moran’s I test statistic allowed testing the null hypothesis of no significant clustering across the entire study region/zones. Moreover, the Anselin local Moran’s index was employed to identify areas with significant neighborhood clustering [22,23].

The following spatial autocorrelation patterns were considered:

- High–high: Positive spatial autocorrelation indicating high-value clustering.
- Low–low: Positive spatial autocorrelation indicating clustering of low values.
- Low–high: Negative spatial autocorrelation indicating that low-value rates are adjacent to high-value rates.
- High–low: Negative spatial autocorrelation indicating that high values are adjacent to low-value rates.
- Not significant: Indicates that there is no spatial autocorrelation.

### Hot spot analysis (Getis OrdGi * statistic)

The Hotspot statistics, using the Gi * statistic, were computed to measure how spatial autocorrelation varies over the study location by calculating Gi * for each area. Z-scores were utilized to determine the statistical significance of clustering in LARC use.

### Spatial interpolation

To predict un-sampled/unmeasured values of LARC use, a spatial interpolation technique was employed. The interpolation method used was Kriging interpolation, which produced a spatial interpolation map based on continuous images of LARC use cases. This process was performed using ArcGIS software to map the clusters and attributes of LARC use [24,25].

### Ethical considerations

Ethical clearance was obtained from the Ethical Review Committee of Wollo University, College of Medicine and Health Sciences. An authorization letter of permission for downloading the 2019 EDHS dataset was obtained from the Demographic and Health Survey (DHS) Institutional Review Board (IRB) of ICF Macro International, USA. Then the EMDHS 2019 dataset was obtained and used with the prior permission of the Central Statistical Agency (CSA) of Ethiopia. This was done firstly by registering for dataset access and writing the title and significance of the study on the website after completing a short registration form. Downloading of datasets was done by using the accessed website at www.measuredhs.com on request with the help of ICF International. The data were only used for this study. It couldn’t be passed to other researchers without the consent of DHS. All DHS data were treated as confidential and no need to identify any household or individual respondent interviewed in the survey. Ethical consent requested during primary data collection was described in the EDHS guide. All the methods were conducted based on the requirement of the relevant guidelines and regulations.

## Results

### Socio-demographic and individual characteristics of the participants

The analysis included a total of 5,743 married women. Among the participants, approximately half (47.74%) were below the age of 30 and 47.76% had their first birth before the age of 18. Only a small proportion (4.05%) of women had received higher education, while a significant majority (51.87%) had no formal education at all. Around 38.37% of the mothers identified themselves as Orthodox followers. Concerning the number of living children, 10.99% of women had not yet had any living children (Table 1).

**Table 1:** Socio-demographic and individual characteristics of the participants who gave birth five years before the survey in Ethiopia.

Variables	Category	Frequency	Percentage
Age of the mothers in complete years	15–19	449	7.81
	20–24	929	16.18
	25–29	1364	23.75
	30–34	1016	17.70
	35–39	901	15.70
	40–44	634	11.04
	45–49	449	7.82
Age of the mothers at first birth	Less than 18 years	2,462	47.76
	18 and above	2,693	52.24
Educational status	No education	2,979	51.87
	Primary	2,078	36.19
	Secondary	453	7.89
	Higher	233	4.05
Parity	0–3	2,972	51.75
	4–5	1,199	20.87
	6 and above	1,572	27.38
No living children	No child	631	10.99
	1–2	1822	31.72
	3–4	1448	25.21
	5 and above	1842	32.08
Preceding birth interval	First birth	588	12.05
	Less than two years	874	17.91
	2–5 years	2550	52.25
	Above five years	868	17.79
Religion	Orthodox	2,203	38.37
	Protestant	1,601	27.87
	Muslim	1,835	31.96
	Others	104	1.81

### Household and community characteristics of the participants

Out of the total participants, 2,178 (37.92%) women were living in households classified below the middle wealth quintile, while 1,225 (21.34%) belonged to the richest wealth quintile. Approximately 382 (6.65%) women reported having fewer than three family members in their households. Interestingly, more than one in ten households (10.27%) was headed by females. Additionally, almost half of the women (48.09%) resided in communities with low levels of female education, and 3,059 (53.27%) lived in communities characterized by high poverty levels (Table 2).

**Table 2:** Household and community characteristics of the participants in Ethiopia.

Variables	Category	Frequency	Percentage
Wealth index	Poorest	1,056	18.38
	Poorer	1,122	19.54
	Middle	1,137	19.79
	Richer	1,203	20.95
	Richest	1,225	21.34
Family size	1–2	382	6.65
	3–5	2628	45.76
	6–7	1656	28.84
	8–10	939	16.34
	11 and above	138	2.4
Household media exposure	Yes	2,189	38.12
	No	3,554	61.88
Community media exposure	Low	2,838	49.42
	High	2,905	50.58
Community female education	Low	2,762	48.09
	High	2,981	51.91
Community poverty	Low	2,684	46.73
	High	3,059	53.27
Place of residence	Urban	1,569	27.31
	Rural	4,174	72.69
Region	Tigray	357	6.21
	Afar	64	1.11
	Amhara	1301	22.66
	Oromia	2240	39.01
	Somali	281	4.89
	Benishangul Gumz	66	1.15
	SNNP	1162	20.23
	Gambela	24	0.42
	Harari	16	0.28
	Addis Ababa	197	3.43
	Dire Dawa	35	0.6

### Spatial distribution of LARC use

**Fig 3:**
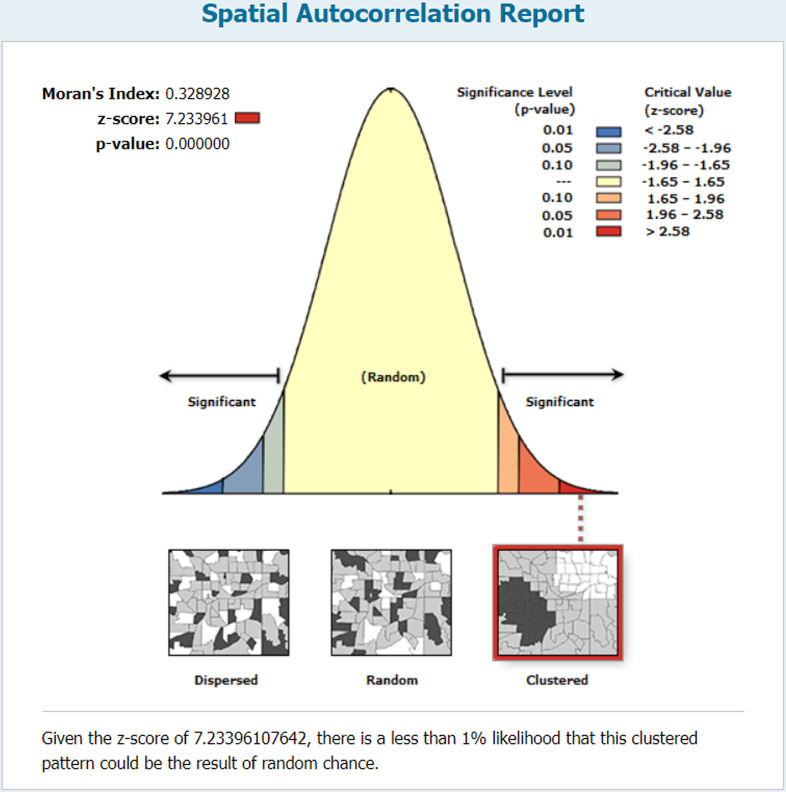
Global spatial auto-correlation (Moran’s I) for LARC use among married women in Ethiopia.

The analysis revealed spatial variation in LARC use across Ethiopia, with a significant Moran’s index of 0.33 (p-value <  0.0001) (Fig 3).

**Fig 4:**
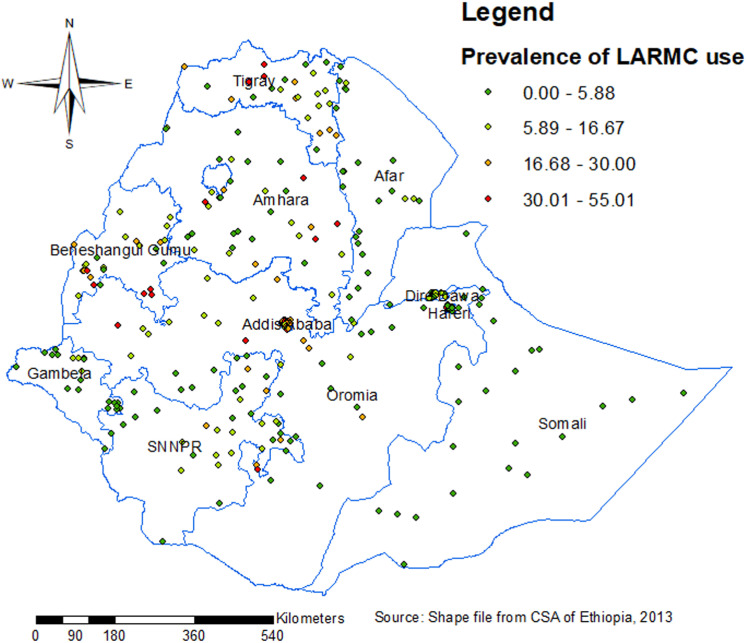
Spatial distribution of LARC use among married women in Ethiopia by different regions.

The spatial distribution of LARC use was mapped using 305 clusters, and high prevalence clusters were identified in Northern and Central Tigray, Eastern parts of SNNP, Eastern Amhara, West and East Benishangul Gumz, and Addis Ababa (Fig 4).

### Cluster and outlier analysis

**Fig 5:**
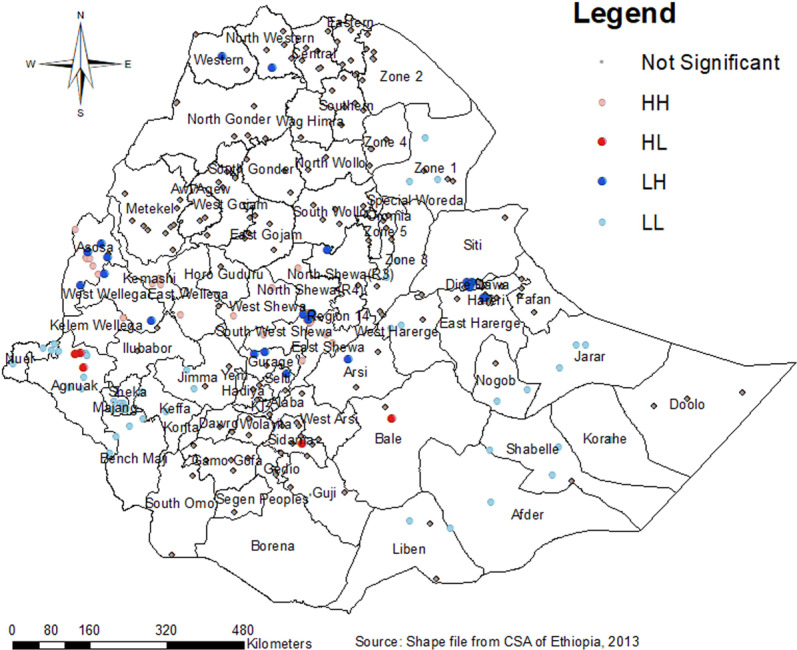
Cluster and outlier analysis (Anselin local Moran’s I) of LARC use among married women in Ethiopia by Zones.

Cluster and outlier analysis were used to identify significant neighborhood clustering. High clustering (HH) indicated a high prevalence of LARC use surrounded by similar characteristics, while low clustering (LL) indicated low prevalence surrounded by similar characteristics. HL and LH clusters represented high prevalence surrounded by low and low prevalence surrounded by high prevalence, respectively. Outliers were found in Dire Dewa, Gurage, Agnuak, Sidama, Bale, Southwest Shewa, and West and Northwestern Tigray (Fig 5).

### Hot spot analysis

**Fig 6:**
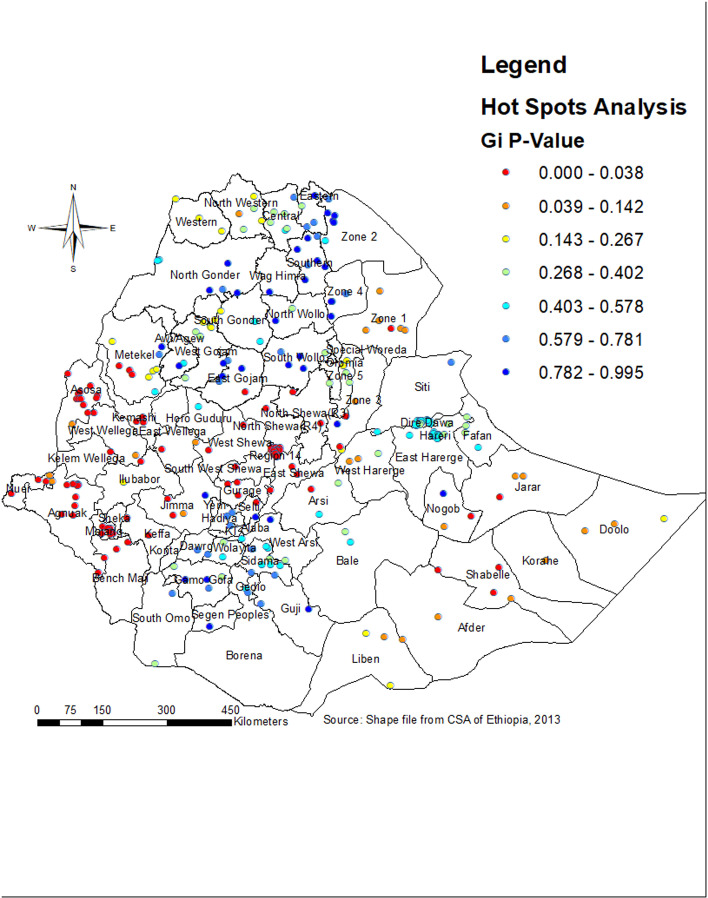
Hot spot analysis for LARC use among married women in Ethiopia by zones.

The Getis-OrdGi * statistics identified hot and cold spots, representing areas with high and low probabilities of LARC use, respectively. The hotspots (red color) were found in Nogob, Shabelle, and Jarar in the Somali region, East Gojam and North Shewa in Amhara region, North Shewa, Southwest Shewa, East Shewa, Jimma and Arsi in Oromia region, Assosa, and Metekel in Benishangul Gumz, Gurage, Sheka, and Keffa in SNNP region, and Anuak in Gambella region (Fig 6).

### Spatial Kriging interpolation

**Fig 7:**
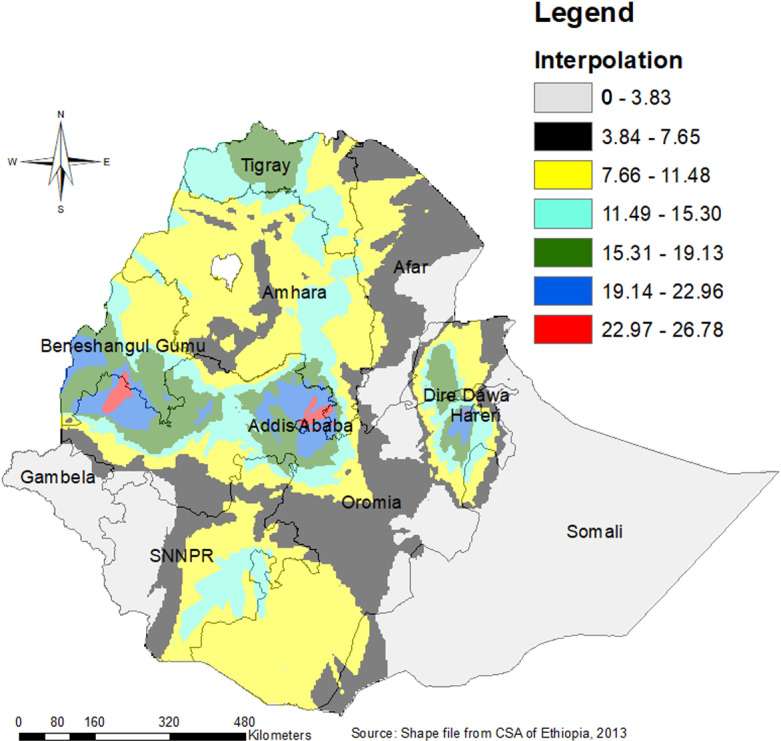
Interpolation for LARC use among married women in Ethiopia by nine geographical regions.

The spatial kriging interpolation analysis predicted regions with a high probability of LARC use. Addis Ababa, Dire Dawa, the Western part of Benishangul Gumz, the Central part of the Harari region, and the Northwestern part of the Oromia region were predicted to have a high probability of LARC use compared to other regions. Conversely, most parts of the Somali region, the majority of Afar and Gambela, and Western parts of SNNP were predicted to have a lower probability of LARC use (Fig 7).

### Factors associated with LARC use (fixed effect)

After adjusting for individual and community-level factors, five variables were found to be statistically significant predictors of LARC use in Ethiopia. These variables were age of the women, educational status of the women, community wealth, community female education, and region. Women aged above 44 years were 72% less likely to use LARC compared to those aged below 20 years [AOR = 0.28, 95% CI: (0.12, 0.64)]. Women with primary education were 1.53 times more likely to use LARC as compared to women with no education [AOR = 1.53, 95% CI: (1.19, 1.97)]. Similarly, women having secondary education were 1.19 times more likely to use LARC as compared to women with no education [AOR = 1.19, 95% CI: (1.32, 2.99)]. In addition, women having higher education were 2.99 times more likely to use LARC as compared to women with no education [AOR = 2.99, 95% CI: (1.82, 4.92)]. Women living in a community with high female education were 1.75 times more likely to use LARC than those in communities with low female education [AOR = 1.75, 95%CI: (1.10, 2.76)]. Additionally, women living in communities with high wealth were 1.92 times more likely to use LARC than those in communities with low wealth [AOR = 1.92, 95% CI: (1.15, 3.2)]. On the other hand, women living in the Somali region were 97% less likely to use LARC compared to women in Dire Dawa [AOR = 0.03, 95% CI: (0.00, 0.33)] (Table 3).

**Table 3:** Bi-variable and multi-variable multi-level binary logistic regression for LARC utilization among married women in Ethiopia.

Variables	Null model (Model 1) ICC = 27.07%	Model 2 AOR (95% CI)	Model 3 AOR (95% CI)
Number of living children			
0	1		
1–2		2.90 (0.51, 16.39)	2.78 (0.49, 15.69)
3–4		3.95 (0.68, 22.97)	3.98 (0.68,23.21)
5 and above		5.36 (0.88, 32.70)	5.57 (0.91, 34.07)
Age at first birth			
Less than 18	1		
18–44		1.18 (0.94, 1.49)	1.22 (0.96, 1.54)
Age of a mother in years			
15–19	1		
20–24		1.03 (0.56, 1.87)	0.98 (0.53, 1.78)
25–29		1.15 (0.63, 2.08)	1.07 (0.59, 1.95)
30–34		0.98 (0.51, 1.87)	0.87 (0.45, 1.66)
35–39		0.98 (0.49, 1.93)	0.84 (0.42, 1.67)
40–44		0.64 (0.31, 1.33)	0.55 (0.26, 1.15)
45–49		0.34 (0.15, 0.78)	0.28 (0.12, 0.64)
Religion			
Orthodox	1		
Protestant		0.77 (0.54, 1.09)	0.95 (0.64, 1.40)
Muslim		0.61 (0.42, 0.90)	0.89 (0.60, 1.33)
Others		0.41 (0.12, 1.37)	0.51 (0.15, 1.74)
Parity			
1–3	1		
4–5		0.97 (0.66, 1.43)	0.99 (0.67, 1.46)
6 and above		0.85 (0.49, 1.47)	0.89 (0.52, 1.54)
Family size			
1–2	1		
3–5		1.25 (0.54, 2.90)	1.23 (0.53, 2.86)
6–7		0.92 (0.38, 2.21)	0.91 (0.38, 2.18)
8–10		1.31 (0.52, 3.25)	1.31 (0.53, 3.27)
11 and above		1.01 (0.30, 3.32)	1.14 (0.34, 3.79)
Household media exposure			
Yes		1.09 (0.84, 1.40)	1.05 (0.81, 1.36)
No	1		
Wealth			
Poorest	1		
Poorer		1.68 (1.14, 2.48)	1.41 (0.95, 2.09)
Middle		1.57 (1.05, 2.32)	1.21 (0.81, 1.81)
Richer		1.40 (0.93, 2.12)	1.02 (0.67, 1.57)
Richest		1.43 (0.88, 2.34)	0.84 (0.49, 1.45)
Educational status of a mother			
Not educated	1		
Primary		1.61 (1.25, 2.06)	1.53 (1.19, 1.97)
Secondary		2.12 (1.41, 3.17)	1.99 (1.32, 2.99)
Higher		3.21 (1.95, 5.26)	2.99 (1.82, 4.92)
Community media exposure			
High			0.96 (0.58, 1.57)
Low	1		
Community wealth			
High			1.92 (1.15, 3.20)
Low	1		
Community female education			
High			1.75 (1.10, 2.76)
Low	1		
Place of residence			
Rural			1.78 (0.97, 3.25)
Urban	1		
Region			
Tigray			0.88 (0.24, 3.26)
Afar			0.15 (0.02, 1.36)
Amhara			0.60 (0.17, 2.18)
Oromia			0.41 (0.12, 1.45)
Somali			0.03 (0.00, 0.33)
Benishangul gumz			1.39 (0.33, 5.88)
SNNP			0.39 (0.11, 1.43)
Gambela	1		0.05 (0.00, 2.35)
Harari			0.70 (0.09, 5.25)
Addis Adaba			1.33 (0.36, 4.92)
Dire Dawa	1		

###  Random effect

The random effect analysis results showed a significant correlation between observations taken from the same cluster, with an Intra-cluster Correlation (ICC) of 27.07%. This indicates that approximately 27% of the variation in the use of LARC can be attributed to the community or cluster level. The analysis further revealed that 33% of the variation in LARC use was explained by the full model.

Additionally, the Median Odds Ratio (MOR) confirmed the impact of community-level factors on LARC use. In the empty model, the MOR was 4.2, indicating variation between communities (clustering) since the MOR was 4.2 times higher than the reference value of 1. However, even after considering all individual and community factors in the full model, the effects of clustering remained statistically significant, but the unexplained community variation in LARC use decreased to a MOR of 2.1 (Table 4).

**Table 4:** Measure of variation for LARC utilization among married women in Ethiopia.

Measures of variation	Null model (model 1)	Model 2	Model 3
Variance	1.22	1.01	0.82
Intra-cluster correlation coefficient (ICC)	27.07	23.51	19.97
Proportionate change in variance (PCV)	Reference	17	33
Median odds ratio (MOR)	4.2	2.6	2.1
Model fitness			
Log-likelihood	-1702.4	-1535.2	-1505.4

## Discussion

This study aimed to analyze the spatial distribution of long-acting reversible modern contraceptive (LARC) use and identify the influencing factors among married women of reproductive age in Ethiopia, using data from the 2019 mini Ethiopian Demographic and Health Survey (EDHS). The result showed that the utilization of LARC varied significantly across different regions in Ethiopia. The factors found to be significantly associated with LARC utilization were educational status, age, region, community wealth, and community level of female education.

Regarding age, the study revealed that women in the age group of 45-49 were less likely to use LARC compared to those in the age group below 20. This finding is consistent with previous studies conducted in Ethiopia [17,26,27]. However, it contrasts with another study conducted in Ethiopia, which reported that women aged 25 and above were more likely to use LARC than those below the age of 25 [28]. Additionally, studies in Sub-Saharan Africa and India showed that women above the age of 35 were more likely to use LARC compared to younger women [8,29]. The possible explanation for the negative association between LARC utilization and women in the age group of 45-49 could be attributed to several factors: Women in this age group are close to menopause, or may be less sexually active. As a result, they may no longer feel the need for long-term contraception. Furthermore, older women might be less likely to be worry for future pregnancies due to various life factors, such as their children reaching adulthood or other family commitments. Consequently, they may perceive a lower need for contraception. In another way, women in this age range might have health concerns or comorbidities that could influence their decision to use certain contraceptive methods, especially long-acting ones. They might prefer less invasive or short-term options [30].

Women who had higher education and who live in a community with high education levels were more likely to use LARC. This finding is similar to studies conducted in Gondar City [31,32] and North-East Ethiopia [33]. There is also a similar report from a study conducted in Kenya [34], Sub-Saharan Africa [35,36] and Nigeria [37]. Educated women, as well as those residing in educated communities, are more likely to choose Long-Acting Reversible Contraceptives (LARC). This preference could be due to their better access to information about family planning, including LARC benefits, and heightened awareness of its effectiveness. Education empowers women to make informed reproductive health decisions, prioritizing their personal and professional goals. Additionally, educated women are more aware of the health risks associated with frequent pregnancies, leading them to opt for LARC to space births and promote maternal and child health. Similarly, women in communities with higher education levels tend to utilize LARC more. In these educated communities, there’s greater awareness and acceptance of family planning methods, including LARC, enhanced access to information and healthcare services facilitates easier adoption of LARC. Moreover, the health-seeking behavior prevalent in educated communities facilitates discussions about family planning options with healthcare providers, thus increasing the likelihood of choosing LARC. Additionally, social norms in educated communities often support family planning and modern contraceptive use [38].

Community wealth was another significantly associated community-level variable. Women who live in a community with high wealth were more likely to utilize LARC. There is also a similar finding from a study done in Ethiopia [39]. However, a study conducted in Nigeria reported that women who reside in a community with medium socio-economic status were less likely to use LARC as compared to women who live in a community with low socio-economic status [40]. The discrepancy might be due to the difference in socio-demographic characteristics, method of analysis, sample size, and study time variation. The possible justification for women who live in a community with high wealth to utilize LARC might be: women should have money for transport and service. The trade-off associated with the time they spent traveling to and from health facilities is also important. They may use that particular time for household activities, farming, or other business-generating activities. For example, in Ethiopia, despite family planning services is free of charge in public health facilities, the cost of transport might be attributable to the use of modern contraception. Moreover, the costs of family planning services in private health facilities might also be related to the use of modern contraceptives. Thus, the cost of transport and family planning service fees in private health facilities might not be important for wealthy families [39]. In addition, women living in communities with better wealth may want to limit their family size to continue their better living standards and well-being [38].

Region was positively and significantly associated with the use of LARC. There is a similar report from a study conducted in Ethiopia [41,42] and India [43]. The use of long-acting family planning methods may vary across different regions of Ethiopia due to a combination of factors: Some regions may be more conservative, leading to resistance or hesitation towards using long-acting methods and regions with higher levels of education and awareness about family planning may have greater acceptance and utilization of long-acting methods. Conversely, areas with limited access to information and education may have lower adoption rates. Likewise, disparities in healthcare infrastructure and access to family planning services can affect the availability and distribution of long-acting contraceptives. Regions with better healthcare facilities are more likely to provide these methods. Religious beliefs can influence family planning decisions. Regions with dominant religious practices that discourage contraceptive use may have lower utilization of long-acting methods. Furthermore, economic conditions in different regions can impact the affordability and availability of long-acting contraceptives. Variations in the implementation of family planning policies and programs across regions have also impact the promotion and distribution of long-acting methods. Similarly, differences in the training and attitudes of healthcare providers regarding long-acting methods can affect their promotion and provision to patients. Remote and rural regions may face geographical and infrastructural challenges that limit access to family planning services, including long-acting methods. In addition, certain regions may have preferences for specific contraceptive methods based on cultural norms or traditional practices, leading to variations in usage and differences in population demographics, such as age structure and fertility rates, can influence the demand for family planning and the choice of contraceptive methods.

## Conclusions

Significant disparities exist in the utilization of long-acting reversible modern contraceptives among women across different regions of the country. This study revealed a statistically significant correlation within cluster, indicating variation between communities in LARC utilization. Both individual and community-level factors played significant roles in determining LARC utilization. Individual factors such as age and educational status were significantly associated, while community-level factors including community female education status, community wealth, and region also showed significant associations. A woman’s educational status not only affected her own health but also influenced the health behaviors of other women in her community. Based on these findings, the study recommends formulating and implementing policies to improve women’s education and awareness of long-acting reversible contraceptives (LARCs) at various population levels. Additionally, efforts should aim to increase and sustain the use of health services across all delivery points. Priority should be given to addressing the needs of the poorest populations, with a focus on strengthening health-seeking behaviors. Regions with low contraceptive use should be targeted with tailored interventions, and culturally sensitive strategies should be developed to promote the adoption of long-acting contraceptive methods.

## Abbreviations

AOR: Adjusted Odds Ratio
CI: Confidence Interval
CSA: Central Statistical Agency
DHS: Demographic and Health Survey
EA: Enumeration Area
EMDHS: Ethiopian Mini Demographic and Health Survey
EFMOH: Ethiopian Federal Minister of Health
EPHI: Ethiopian Public health Institute
GTP: Growth and Transformation Plan
ICC: Intra-class Correlation Coefficient
ICF: International Classification of functioning
LARC: Long Acting Reversible Contraceptive
MOR: median odds ratio
SDG: Sustainable Development Goals
PCV: Proportional change in Variance
PHC: Population and Housing census
WHO: World Health Organization
